# A Vectorial Capacity Product to Monitor Changing Malaria Transmission Potential in Epidemic Regions of Africa

**DOI:** 10.1155/2012/595948

**Published:** 2012-01-26

**Authors:** Pietro Ceccato, Christelle Vancutsem, Robert Klaver, James Rowland, Stephen J. Connor

**Affiliations:** ^1^The International Research Institute for Climate and Society, The Earth Institute, Columbia University, Lamont Campus, 61 Route 9W, Monell Building, Palisades, NY 10964-8000, USA; ^2^U.S. Geological Survey (USGS) Earth Resources Observation and Science (EROS) Center, Sioux Falls, SD 57198, USA; ^3^School of Environmental Sciences, University of Liverpool, Liverpool L69 3BX, UK

## Abstract

Rainfall and temperature are two of the major factors triggering malaria epidemics in warm semi-arid (desert-fringe) and high altitude (highland-fringe) epidemic risk areas. The ability of the mosquitoes to transmit *Plasmodium* spp. is dependent upon a series of biological features generally referred to as vectorial capacity. In this study, the vectorial capacity model (VCAP) was expanded to include the influence of rainfall and temperature variables on malaria transmission potential. Data from two remote sensing products were used to monitor rainfall and temperature and were integrated into the VCAP model. The expanded model was tested in Eritrea and Madagascar to check the viability of the approach. The analysis of VCAP in relation to rainfall, temperature and malaria incidence data in these regions shows that the expanded VCAP correctly tracks the risk of malaria both in regions where rainfall is the limiting factor and in regions where temperature is the limiting factor. The VCAP maps are currently offered as an experimental resource for testing within Malaria Early Warning applications in epidemic prone regions of sub-Saharan Africa. User feedback is currently being collected in preparation for further evaluation and refinement of the VCAP model.

## 1. Introduction

Malaria is a major public health threat to the African continent and its control is critical to achieving the Millennium Development Goals in this region [1].

Although considerable progress has been made to reduce the malaria burden in sub-Saharan Africa by introducing control measures such as the provision of insecticide-treated mosquito nets, indoor residual spraying, and easier access to effective antimalarial drugs [2], malaria epidemics continue to occur. Periodic epidemics of malaria are a major public health problem for many sub-Saharan African countries. Populations in epidemic-prone areas have a poorly developed immunity to malaria and the disease remains life threatening to all age groups [3]. The impact of epidemics could be minimized through prediction, improved prevention through timely vector control, and deployment of appropriate control measures. The implementation of a Malaria Early Warning System enables regional health ministries to focus on epidemiological surveillance and be better prepared to take necessary actions.

Rainfall and temperature anomalies are two of the major environmental factors triggering epidemics in warm semi-arid and altitude areas. Increases in epidemics often occur in these regions after excessive rains or increases in temperature [4, 5]. The ability of the mosquitoes to transmit *Plasmodium *spp. is dependent upon a series of biological features, which all together are generally referred to as vectorial capacity. Vectorial capacity (VCAP) has been defined as the daily rate at which future inoculations could arise from a currently infected case [6]. It is generally used as a convenient way to express malaria transmission risk or the receptivity of an area to malaria [7]. While vectorial capacity does not take into account parasite availability in the human (intermediate host) population, it is considered to be analogous to the environmental-biological driving force underpinning the transmission potential in an area.

The vectorial capacity model has been expanded to enable temperature and rainfall to drive the model [8, 9].

**Figure 1:**
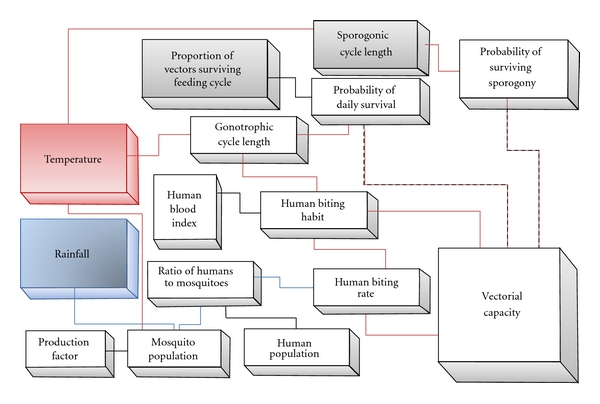
Diagrammatic representation of “expanded” vectorial capacity (VCAP) Model.

The expanded vectorial capacity model considers the influence of rainfall and temperature variables on malaria transmission patterns by including the impact they have on the bionomics of the anopheline vector (feeding frequency, gonotrophic period, larval development rate, and survival) and the parasite extrinsic incubation period (sporogony) in its mosquito (definitive) host (Figure 1).

In this study, we have evolved the vectorial capacity model by integrating it with two remotely sensed products to monitor rainfall and temperature based on satellite images. The remotely sensed products provide information at high spatial and temporal resolutions. The resulting vectorial capacity model is provided as an Early Warning System to help the malaria control community be prepared and to enable them to provide the timely response needed to save lives.

## 2. Data and Method

Vectorial capacity is a series of biological features that determine the ability of mosquitoes to transmit *Plasmodium. *It is defined as the daily rate at which future inoculations could arise from a currently infected case [6] and it is generally used as a convenient way to express malaria transmission risk or the receptivity of an area to malaria [7]. For the purposes of this study. The VCAP is adapted from the Macdonald model [10] which is summarized in the formula for *Z*_0_, the basic reproduction rate of malaria, as follows:

(1) $$Z_{0}=\frac{-\left(ma^{2}b\right)p^{n}}{r\left[\ln\left(p\right)\right]}=\left(\frac{b}{r}\right)V,$$

where *b* represents the proportion of those *anophelines* with sporozoites in their salivary glands which are actually infective, *r* is used to represent the proportion of infected hosts per day, and VCAP is the “vectorial capacity” of malaria, given by

(2) $$\text{VCAP}=\frac{-\left(ma^{2}\right)p^{n}}{\ln\left(p\right)}.$$

Parameter *p* is the probability of a mosquito surviving through one whole day and *n* is the extrinsic incubation period of malaria parasites or ‘‘the time taken for completion of the extrinsic cycle”.

Macdonald derived the basic reproduction rate *Z*_0_ as an estimate of the average number of secondary cases arising in a very large population of completely susceptible humans at risk following the introduction of a single primary case. *Z*_0_ = 1 was defined as the transmission threshold: for values of *Z*_0_ above 1, malaria cases will propagate, while, for values of *Z*_0_ below 1, the disease will recede.

Rainfall and temperature are used as inputs to the model because they have an impact on vectorial capacity. Temperature has an effect on both the vector and the parasite. For the vector, it affects the juvenile development rates, the length of the gonotrophic cycle, and survivorship of both juvenile and adult stages with an optimal temperature and upper and lower lethal boundaries. For the parasite, it effects the extrinsic incubation period [11]. *Plasmodium falciparum* (the dominant malaria parasite in Africa) requires warmer minimum temperature than *Plasmodium vivax*. This helps to account for the geographic limits of malaria transmission for this particular species in Africa [12]. At 26°C the extrinsic incubation period of this malaria species is about 9-10 days whereas at 20–22°C it may take as long as 15–20 days. In highland areas, where cold temperatures preclude vector and/or parasite development during part/or all of the year, increased prevalence rates may be closely associated with higher than average minimum temperatures [13].

The association between rainfall and malaria epidemics has been recognized for many dekades [14] but, while increasing precipitation may increase vector populations in many circumstances by increasing available anopheles breeding sites, excessive rains may also have the opposite effect by flushing out small breeding sites, such as ditches or pools [15] or by decreasing the temperature, which in regions of higher altitude can stop malaria transmission. Different malaria vectors use a variety of sites in which to lay their eggs (irrigation canals, tire ruts, mangrove swamps, pools, etc.) as long as the water is clean, not too shaded and, for most species, relatively still. In many semiarid areas these sites are only widely available with the onset of the seasonal rains unless dry season irrigation is undertaken.

In Africa, the spatial distribution of weather stations is often limited and the dissemination of rainfall and temperature data is variable, therefore limiting their use for real-time applications. Compensation for this paucity of information can be obtained by using satellite-based methods to monitor rainfall and air temperature. Based on previous validations of rainfall estimate products with rain gauge data [16] we decided to use the satellite products derived from Tropical Rainfall Measuring Mission (TRMM) sensor. The TRMM products [17] are available on a three-hour basis from 1998 at 0.25 deg spatial resolution and have been shown to provide a better spatial and temporal estimation of rainfall in Africa [16] than most other rainfall estimate products.

The derivation of near-surface air temperature (*T*_*a*_) from land surface temperature (*T*_*s*_) derived from satellite is far from straightforward. Recent studies conducted by Vancutsem et al. [18] showed that night *T*_*s*_ data derived from the Moderate Resolution Imaging Spectroradiometer (MODIS) provide a good estimation of minimum *T*_*a*_ over different ecosystems. We opted to use MODIS (on board of the Aqua satellite) night time *T*_*s*_ available on an 8 day basis at 1 km spatial resolution to estimate minimum *T*_*a*_ and to compute the VCAP model.

The USGS EROS Center combined the temperature derived from MODIS night *T*_*s*_ on an 8-day basis and the TRMM rainfall downscaled to 1 km spatial resolution to produce a 1 km VCAP map every 8 days specifically for the epidemic regions of sub-Saharan Africa. In (2), the two raster images, MODIS night time (*T*_*s*_) and rainfall (TRMM), are integrated using an ArcView script written in Avenue object-oriented programming language as follows:

(3) $$\;\text{VCAP}=\frac{-\left(ma^{2}\right)p^{n}}{\ln\left(p\right)},$$

where *m* = 10.0  ∗  TRMM, *a* = 0.7/gonotrophic, gonotrophic = [36.5/(*T*_*s*_ + 2.0 − 9.9)] + 0.5,  *p* = 0.5^(1.0/gonotrophic)^, and *n* = 111.0/{[2.0∗(36.5/*T*_*s*_ + 2.0 − 9.9)/gonotrophic] + *T*_*s*_ − 18.0}.

The coefficients used in the VCAP equation are at this stage not optimized to specific regions. The variability in VCAP is only driven by the *T*_*s*_ and rainfall. This is a first attempt to spatially map risk of malaria transmission based on a vectorial capacity model.

**Figure 2:**
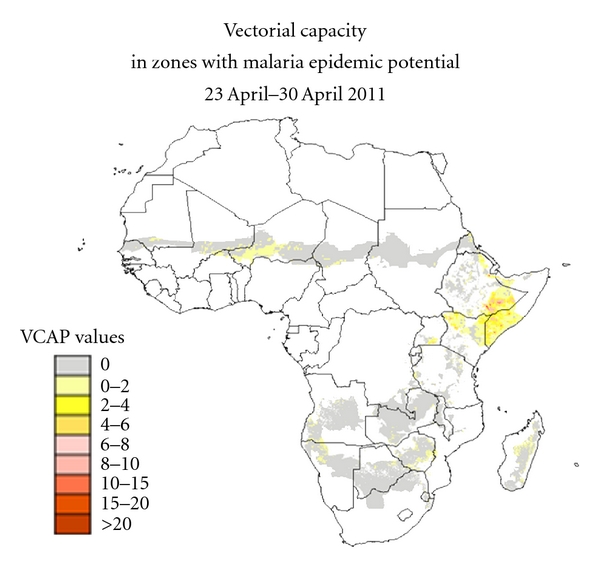
Vectorial capacity map produced by USGS EROS Center. The VCAP values are provided for the epidemic zones of Africa at 1 km spatial resolution. The map has been updated every 8 days since January 2004.

The product (Figure 2) is made available on a regular basis for the period from January 2004 to present on the FEWS NET Africa Data Portal website: http://earlywarning.usgs.gov/fews/africa/web/imgbrowses2.php?extent=afvc.

The existence of this online VCAP is publicized and its use and validation by control services and researchers is encouraged. Validation of the VCAP product was performed using malaria data provided by (i) the National Malaria Control Program, Ministry of Health, Eritrea, and (ii) the *Service de Lutte contre le Paludisme*, Antananarivo, Madagascar. Results are presented here next. 

## 3. Results and Discussion

### 3.1. Eritrea

**Figure 3:**
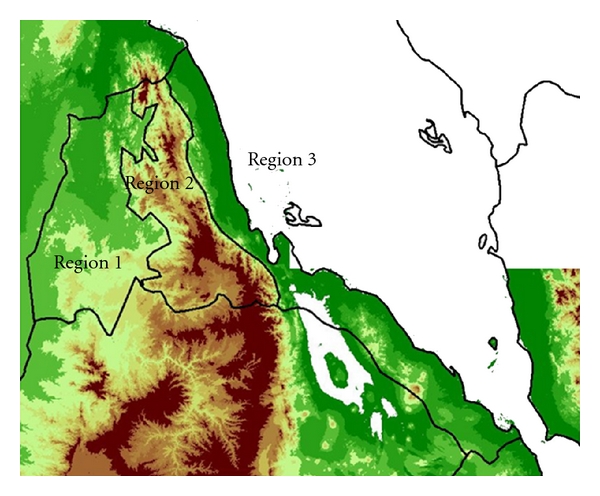
Eritrea map showing the topography of the country with three regions: Region 1 is characterized by low land with high malaria incidence (peak in October), Region 2 is characterized by a mountain area with low malaria incidence (peak in October), and Region 3 is characterized by low land area in the Red Sea coast with low malaria incidence values (peak in January).

Eritrea has a successful malaria control program which has resulted in a substantial decrease in the number of malaria cases from 2000 to 2009 [2]. However, the country is still susceptible to malaria increases during certain climatic conditions (increase of temperature in the highland or increased rainfall in the lowland). Ceccato et al. [19] identified regions in the western Eritrea with high incidence values of malaria that peaked in October, areas in the mountain region with low malaria incidence values that peaked in October, and regions in the eastern Eritrea with low malaria incidence values that peaked in January. The three different regions were selected to analyze the evolution of VCAP in relation to malaria incidence, temperature, and rainfall (Figure 3).

**Figure 4:**
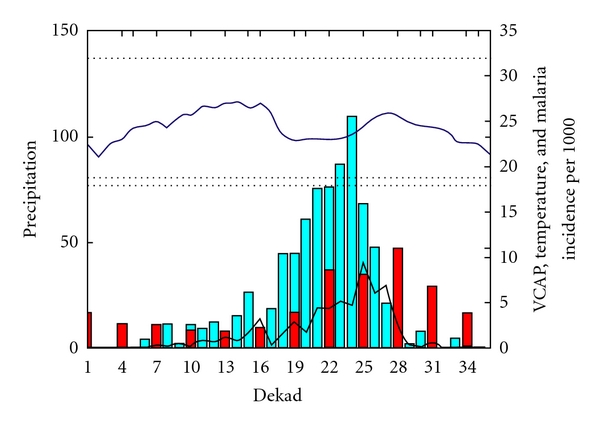
the black line represents the VCAP values (between 0 and 35), the blue bar represents rainfall (mm/dekad from 0 to 150 mm), the blue line represents temperature (between 18°C and 30°C), and the Red bar represents malaria incidence per 1000 habitants.

The evolution of VCAP in relation to temperature, rainfall and malaria incidence for Region 1 (Figure 4) indicates that, while the temperature is not a limiting factor temperature above 18 and below 32° and rainfall peaks in September (22 to 25 dekades), the VCAP increases from dekad 10 and reaches its peak in dekad 25. The malaria incidence values follow the increase of VCAP peaking in dekad 28 (3 dekads after the peak of VCAP).

**Figure 5:**
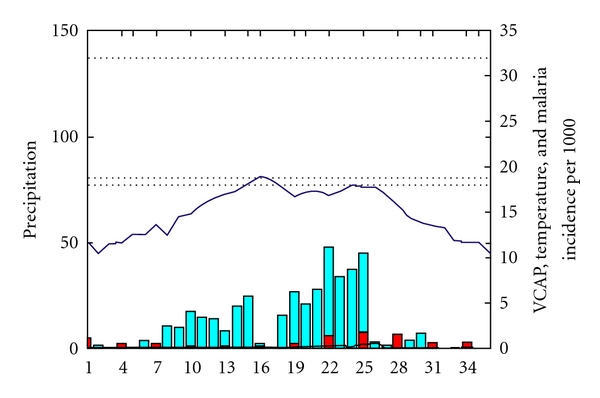
Region 2: the black line represents the VCAP values (low values), the blue bar represents rainfall (mm/dekad with values below 50 mm), the blue line represents temperature (below 18°C), and the red bar represents malaria incidence per 1000 habitants.

The evolution of VCAP in relation to temperature, rainfall, and malaria incidence for Region 2 (Figure 5) indicates that the temperature is the limiting factor (temperature always below 18°C). While rainfall peaks in September (22 to 25 dekads), the VCAP correctly indicates that the risk for malaria transmission is low since the conditions for malaria transmission are not favorable due to low temperature. The malaria incidence values are also low as would be expected in this environment.

**Figure 6:**
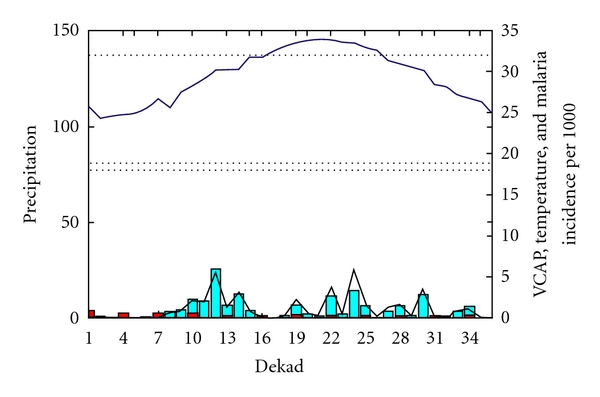
Region 3: the black line represents the VCAP values (low values reaching 5), the blue bar represents rainfall (mm/dekad with values below 25 mm), the blue line represents temperature (high temperature above 25°C), and the red bar represents malaria incidence per 1000 habitants.

The evolution of the VCAP in relation to temperature, rainfall and malaria incidence for Region 3 (Figure 6) indicates that the temperature is not the limiting factor (temperature always above 25°C), but the rainfall is (values below 20 mm/dekad). The VCAP values correctly indicate that the risk for malaria transmission is low since the conditions for malaria transmission are low due to low rainfall. The malaria incidence values are also low as would be expected in this environment.

These limited time series of malaria incidences covering a period during which control measures have been very effective and malaria number decreases do not allow us to demonstrate yet that the extended VCAP can predict interannual variation (i.e., epidemics). The product shows promising results as a tool to monitor the risk of malaria. However, the model should be evaluated further and refined with longer time series and ideally for regions where control measures have not been implemented to fully validate the VCAP relationship with epidemic occurrences.

### 3.2. Madagascar

**Figure 7:**
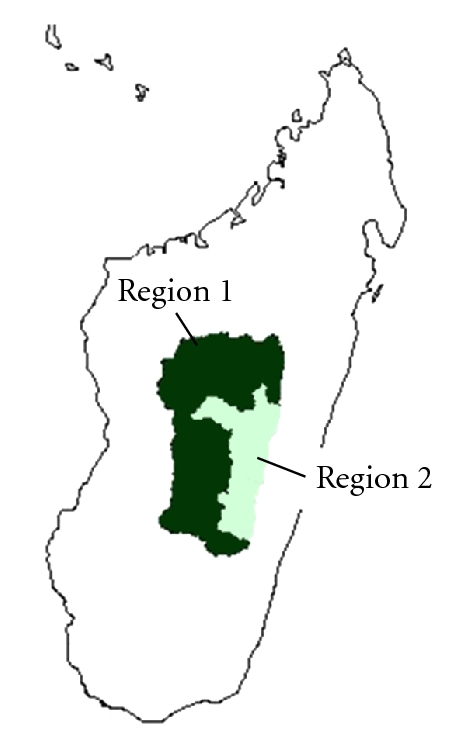
Regions in Madagascar. Region 1 is characterized by high malaria incidence values peaking in March. Region 2 is characterized by low malaria incidence values due to high altitude.

Similar to Eritrea, Madagascar has a successful malaria control program which has substantially decreased the number of malaria cases during the period 2004 to 2009. However, in the central mountain region we identified a region with high incidence values of malaria peaking in March (Region 1 in Figure 7) and a region in the highlands with low malaria incidence values (Region 2 in Figure 7). Based on malaria incidence values for the period 2004 to 2006, we analyzed the relationship between VCAP values and malaria incidence values, rainfall, and temperature (Figure 7).

**Figure 8:**
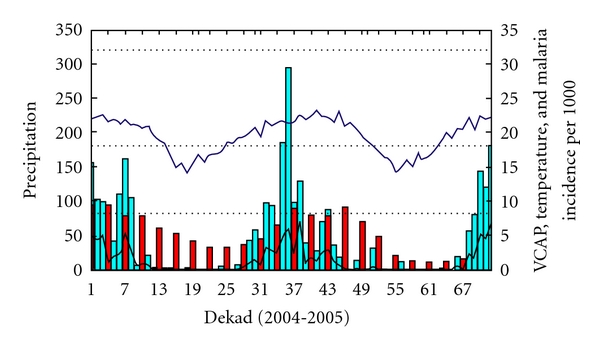
Region 1: the black line represents the VCAP values (values from 0 to 8), the blue bar represents rainfall (mm/dekad with values reaching 300 mm), the blue line represents temperature (temperature between 15 and 25°C), and the red bar represents malaria incidence per 1000 habitants.

The evolution of VCAP in relation to temperature, rainfall, and malaria incidence for Madagascar Region 1 (Figure 8) indicates that the temperature is not the limiting factor (temperature is always above 18°C except during the dekads 14 to 25 and 51 to 62 which corresponds to the end of April 2004 to August 2004 and from April 2005 to August 2005). The rainfall, however, is a limiting factor during the same period but increases during the months of September to March peaking in January. The VCAP values follow the rainfall pattern during the rainy season in Madagascar and are followed, in turn, by the increase of malaria incidence values.

**Figure 9:**
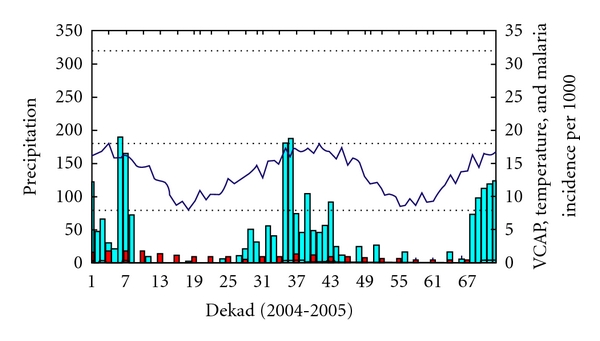
Region 2: the black line represents the VCAP values (very low values close to 0), the blue bar represents rainfall (mm/dekad with values reaching 200 mm), the blue line represents temperature (temperature below 18°C), and the red bar represents malaria incidence per 1000 habitants.

The evolution of the VCAP in relation to temperature, rainfall, and malaria incidence for Madagascar Region 2 (Figure 9) indicates that the temperature is the limiting factor (temperature always below 18°C). Despite good rainfall conditions, the VCAP is low, as are the malaria incidence values. This is unsurprising since the unfavorable temperature conditions for the mosquito would be expected to result in low malaria incidence values.

The results obtained for Eritrea and Madagascar show the potential benefit in using VCAP to assess the risk of malaria incidence in different regions where either temperature or rainfall can be limiting factors. Although the malaria dataset was rather limited, it did allow us to differentiate regions with low VCAP values and subsequently low malaria incidence values and regions with high VCAP values experiencing high malaria incidence values. The relationship between VCAP and malaria incidence values that we obtained is promising but should be tested further to complete the validation process.

The VCAP maps are currently being offered as an experimental resource to be tested within Malaria Early Warning applications in epidemic-prone regions of sub-Saharan Africa. The product shows promising results as a tool to monitor the risk of malaria in Eritrea and Madagascar. However, we encourage and solicit user feedback so that further evaluation can be conducted and the application can be refined in a future evolution of the model.

## 4. Conclusions

Access to frequently updated VCAP information is an important requirement for the development of integrated early warning systems for malaria. The VCAP maps have been developed primarily for application in warm semiarid and/or high-altitude regions where rainfall and/or temperature are the main determinant of epidemic outbreaks. The analysis of VCAP in relation to rainfall, temperature, and malaria incidence data in Eritrea and Madagascar shows that the VCAP correctly tracks the risk of malaria both in regions where rainfall is the limiting factor and in regions where temperature is the limiting factor. The VCAP maps are currently being offered as an experimental resource for testing within Malaria early warning applications in epidemic-prone regions of sub-Saharan Africa and highlands. The product shows promising results as a tool to monitor the risk of malaria. However, user feedback is solicited so that the model can be evaluated further and refined at a later date.
